# *Bartonella* Prevalence and Genome Sequences in Rodents in Some Regions of Xinjiang, China

**DOI:** 10.1128/aem.01964-22

**Published:** 2023-03-23

**Authors:** Ai-Ling Xu, Yan-Fang Chen, Long Mu, Peng-Bo Liu, Jun Wang, Rui-Xiao Li, Dong-Mei Li

**Affiliations:** a State Key Laboratory of Infectious Disease Prevention and Control, Collaborative Innovation Center for Diagnosis and Treatment of Infectious Diseases, Department of Vector Biology and Control, National Institute for Communicable Disease Control and Prevention, Chinese Center for Disease Control and Prevention, Beijing, People’s Republic of China; b Institute for Agricultural Sciences of 10th Division of Xinjiang Production and Construction Corps, Beitun City, Xinjiang Uygur Autonomous Region, People’s Republic of China; c School of Public Health, Cheeloo College of Medicine, Shandong University, Jinan, People’s Republic of China; University of Nebraska—Lincoln

**Keywords:** *Bartonella*, genome sequencing, phylogenetic tree, average nucleotide similarity

## Abstract

In this study, we investigated *Bartonella* infection and its genetic diversity in rodents in Beitun, Xinjiang Uygur Autonomous Region, China. Small mammals were captured using snap traps at four sampling sites in 2018. Spleen and liver tissues were collected and cultured to isolate *Bartonella* strains. Whole-genome sequencing was performed on the strains identified as *Bartonella* by *gltA* gene PCR, and the average nucleotide identity (ANI) of the genomes was calculated by using FastANI v1.33. Phylogenetic trees were constructed for the samples positive for *Bartonella* spp. by the *gltA* PCR assay based on 1,290-bp *gltA* genes, 2,903-bp *rpoB* genes, and core-genome single nucleotide polymorphisms (SNPs). Among 66 rodents, 11 were positive for *Bartonella*, with an infection rate of 16.67%. The rodent infection rates in different tissues (χ^2^ = 2.133; *P* = 0.242), species (χ^2^ = 9.631; *P* = 0.141), and habitats (χ^2^ = 4.309; *P* = 0.312) did not show statistical differences. *Bartonella* spp. isolated from the rodents were phylogenetically divided into six clades (two different *Bartonella* species were detected in two rodents). By comparing phylogenetic trees based on *gltA* genes, *rpoB* genes, and SNPs, we found that the topological structures of several evolutionary trees are different. However, the *Bartonella* strains isolated in this study were clustered into six clusters in different phylogenetic trees. Broad distributions and high genetic diversity of *Bartonella* strains were observed among rodents in Beitun, Xinjiang.

**IMPORTANCE** Rodent-borne *Bartonella* species have been associated with zoonotic diseases. *Bartonella* species such as Bartonella elizabethae, Bartonella grahamii, and Bartonella tribocorum can cause disease in humans. Humans can be infected by blood-sucking arthropods through the scratches and bites of an infected reservoir host or via contact with infectious rodents. Xinjiang is one of the provinces with the most abundant species of *Bartonella* in China, but there are few reports about the prevalence of *Bartonella* in the Beitun area. This research aims to investigate the occurrence and prevalence of *Bartonella* infection in rodents at these sampling sites and provide a basis for the prevention and control of rodent *Bartonella* species in Beitun and the surrounding areas of Xinjiang.

## INTRODUCTION

The genus *Bartonella* was previously classified as *Bartonella*, *Grahamella*, *Rochalimaea*, and alphaproteobacteria. These microorganisms are aerobic, Gram-negative, facultative intracellular parasitic, and slow-growing pleiomorphic organisms (1, 2). Bartonella bacilliformis was the only member of the *Bartonella* genus before 1991. The number of members of the genus *Bartonella* has increased since Brenner and colleagues confirmed the widespread belief that species of the genus *Rochalimaea* were more correctly placed into the genus *Bartonella* (2). At present, the genus *Bartonella* contains 40 validated species and subspecies (LPSN [List of Prokaryotic Names with Standing in Nomenclature] [https://lpsn.dsmz.de/]), including over 20 species that have been isolated in wild rodents (3). A wide variety of mammals are suspected reservoir hosts of *Bartonella* spp., such as ruminants, carnivores, and rodents. Among small mammals, bats and rodents have the highest levels of diversity of *Bartonella* spp. (4). Some rodent-adapted *Bartonella* species have been reported to be involved in human infections (Bartonella grahamii, *B. elizabethae*, and *B. washoensis*, etc.) (5). Since many rodents live in proximity to humans, human-rodent contact is inevitable. With the development of the economy and original environments, the exposure of people to diseased host animals and vectors increases, as do the risk and extent of infection with *Bartonella* (6).

As the largest province in China, Xinjiang borders eight countries, including Russia, Kazakhstan, Kyrgyzstan, Tajikistan, Pakistan, Mongolia, India, and Afghanistan. *Bartonella* infections in rodents have been reported in at least four neighboring countries (7–9). Previous studies have confirmed that Xinjiang is one of the provinces with the most abundant species of *Bartonella* in China (10). However, there are only a few studies on *Bartonella* infections in rodents in Xinjiang, and most of them are concentrated in port areas. Beitun is a main traffic route to Russia, Kazakhstan, Mongolia, and other places; it has many tourist attractions because of its special history and geographic position. Therefore, we conducted a baseline survey of the occurrence and prevalence of rodent *Bartonella* infections in Beitun, Xinjiang, China. The potential risk for the transmission of rodent-borne *Bartonella* was evaluated.

## RESULTS

### Rodent trapping.

**FIG 1:**
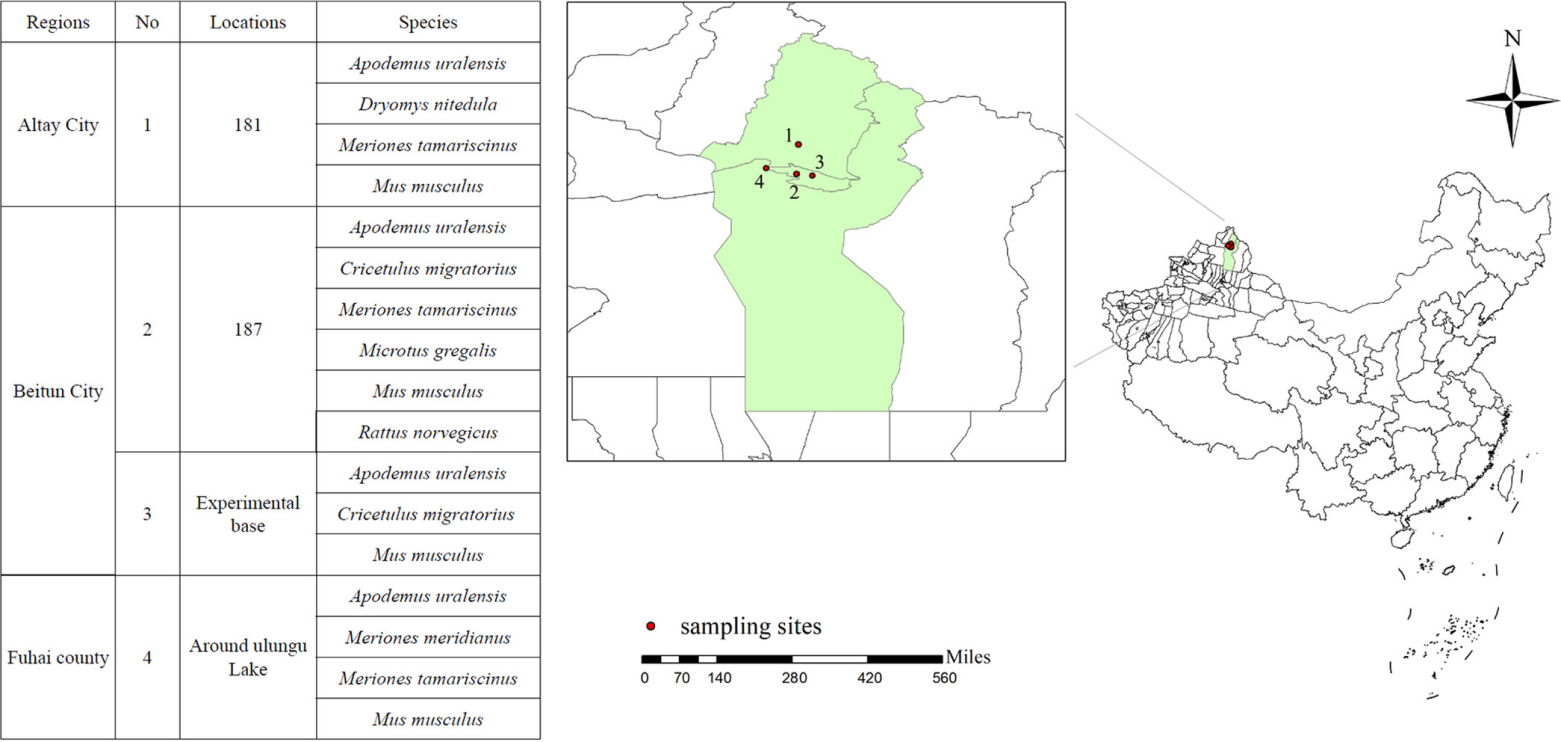
Locations of the sampling sites for rodent collection in Beitun and surrounding areas of Xinjiang (different locations are indicated by numbers; 1 to 4 indicate the sampling sites). The map was made by using ArcGIS software (version 10.5; ESRI Inc., Redlands, CA, USA). 181, 181st regiment of the Tenth Division of Xinjiang Production and Construction; 187, 187th regiment of the Tenth Division of Xinjiang Production and Construction.

A total of 66 rodents belonging to 8 species and 7 genera from 3 rodent families were captured at 4 sampling sites (Fig. 1). Thirty-four rodents were trapped in an *Elaeagnus angustifolia* plantation, five rodents were trapped in a shelterbelt forest, nine rodents were trapped in tamarisk trees, three rodents were trapped in grassland, and six rodents were trapped in a semidesert. *Apodemus uralensis* was the most prevalent animal species and accounted for 46.97% of the rodents (*n* = 31), followed by Rattus norvegicus (*n* = 10), Mus musculus (*n* = 9), *Meriones tamariscinus* (*n* = 7), *Cricetulus migratorius* (*n* = 5), and *Meriones meridianus* (*n* = 2). One *Dryomys nitedula* rodent and one *Microtus gregalis* rodent were captured.

### *Bartonella* prevalence and analysis of host composition.

The prevalence of *Bartonella* in rodents was 16.67% (11/66), but there were no significant variations in infection rates (χ^2^ = 9.631; *P* > 0.05 [by Fisher’s exact test]) among the 8 species (Table 1). Thirteen *Bartonella* isolates were established from tissue samples of 66 rodents in total, including 4 (6.1%) from 66 liver and 9 (13.6%) from 66 spleen samples, and there were no significant variations in infection rates (χ^2^ = 2.133; *P* > 0.05 [by a chi-square test]) (Table 2). The culture positivity rates for the *Elaeagnus angustifolia* plantation, tamarisk trees, and the semidesert habitat were 17.6% (6/34), 44.4% (4/9), and 16.7% (1/6), respectively. Animals captured in two habitats, the shelterbelt forest and grassland, did not develop *Bartonella* infection. There was no statistically significant difference (χ^2^ = 4.309; *P* > 0.05 [by Fisher’s exact test]) in the positivity rates for the five habitats.

**TABLE 1:** *Bartonella* infection rates in liver and spleen tissues collected from rodents in Xinjiang, China

Rodent species	No. of samples	No. of positive samples	Positivity rate (%)
*Apodemus uralensis*	31	6	19.4
*Cricetulus migratorius*	5	1	20.0
*Dryomys nitedula*	1	0	0
*Microtus gregalis*	1	0	0
*Meriones meridianus* ^*a*^	2	1	50.0
Mus musculus	9	0	0
*Meriones tamariscinus*	7	3	42.9
Rattus norvegicus	11	0	0

a Endemic species in China.

**TABLE 2:** Distribution and species identification of *Bartonella* isolates in rodent tissues

Sample ID	Rodent species	Culture result^*b*^		Optimal match with the 320-bp *gltA* gene fragment (% similarity)	Optimal match with the full-length *gltA* gene (% similarity)	Optimal match with the full-length *rpoB* gene (% similarity)	Most closely related type strain (ANI [%])
		Spleen	Liver				
AU5XJBT	*Apodemus uralensis*	0	1	Bartonella tribocorum B29906 (100)	*Bartonella kosoyi* Tel Aviv (95.29)	Bartonella tribocorum CIP 105476 (96.05)	*B. tribocorum* CIP 105476 (93.31)
AU15XJBT	*Apodemus uralensis*	1	1	Bartonella tribocorum B29906 (100)	*Bartonella kosoyi* Tel Aviv (95.29)	Bartonella tribocorum CIP 105476 (96.10)	*B. tribocorum* CIP 105476 (93.29)
AU16XJBT	*Apodemus uralensis*	1	1	Bartonella tribocorum B29906 (100)	*Bartonella kosoyi* Tel Aviv (95.29)	Bartonella tribocorum CIP 105476 (96.05)	*B. tribocorum* CIP 105476 (93.30)
AU4XJBT	*Apodemus uralensis*	1	1	Bartonella grahamii Far East IV (100)	Bartonella grahamii as4aup (99.46)	Bartonella grahamii as4aup (99.64)	B. grahamii NCTC12860 (97.85)
AU18XJBT	*Apodemus uralensis*	0	1	Bartonella grahamii CL70QHWL (97.81)	Bartonella grahamii as4aup (97.69)	Bartonella grahamii as4aup (97.64)	B. grahamii A1JPB (94.60)
CM31XJBT	*Cricetulus migratorius*	1	1	Bartonella grahamii CL70QHWL (99.69)	Bartonella grahamii as4aup (98.61)	Bartonella grahamii as4aup (97.71)	B. grahamii ATCC 700132 (94.44)
AU55XJBT	*Apodemus uralensis*	1	1	Bartonella elizabethae B29908 (100)	Bartonella elizabethae NCTC12898 (96.22)	Bartonella elizabethae NCTC12898 (96.39)	*B. elizabethae* Re6043vi (93.22)
ML69XJBT	*Meriones tamariscinus*	1	0	“*Candidatus* Bartonella gerbillinarum” MM85QHGEM (100)	Bartonella alsatica (90.51)	Bartonella taylorii IBS296 (91.26)	*B. florencae* R4 (86.12)
ML70XJBT.G	*Meriones tamariscinus*	0	1	“*Candidatus* Bartonella gerbillinarum” MM85QHGEM (99.69)	Bartonella alsatica (90.28)	Bartonella taylorii IBS296 (91.04)	*B. florencae* R4 (85.95)
ML71XJBT	*Meriones tamariscinus*	1	1	“*Candidatus* Bartonella gerbillinarum” MM85QHGEM (100)	Bartonella alsatica (90.51)	Bartonella taylorii IBS296 (91.26)	*B. florencae* R4 (86.12)
MM73XJBT	*Meriones meridianus* ^*a*^	1	0	“*Candidatus* Bartonella gerbillinarum” MM85QHGEM (100)	Bartonella alsatica (90.51)	Bartonella taylorii IBS296 (91.26)	*B. florencae* R4 (86.08)
ML70XJBT	*Meriones tamariscinus*	1	0	*Bartonella krasnovii* FN12_1 (98.75)	*Bartonella krasnovii* 75A_4b (97.76)	*Bartonella krasnovii* 51A_7 (97.04)	*B. krasnovii* B714 (94.68)
MM73XJBT.G	*Meriones meridianus* ^*a*^	0	1	*Bartonella krasnovii* FN12_1 (98.75)	*Bartonella krasnovii* 75A_1b (97.76)	*Bartonella krasnovii* 51A_7 (96.92)	*B. krasnovii* B3512 (94.61)

a Endemic species in China.

b 0, negative culture; 1, positive culture.

### Genetic identification and phylogenetic relationships of rodent *Bartonella* isolates.

Initial screening of *Bartonella* DNA was performed using *gltA* PCR. DNA sequence homology analysis and phylogenetic analysis indicated that six *Bartonella* species were detected in the spleens and livers of rodents. Different species of *Bartonella* were isolated from the spleens and livers of animals 70 and 73 (strains ML70XJBT and MM73XJBT, respectively). B. grahamii (1 isolate), B. grahamii-like (2 isolates), *B. tribocorum*-like (3 isolates), *B. elizabethae*-like (1 isolate), and *B. krasnovii*-like (2 isolates) isolates and isolates of unknown genotypes (4 isolates) were obtained from *Apodemus uralensis*, *Cricetulus migratorius*, *Meriones tamariscinus*, and *Meriones meridianus* from Beitun, Xinjiang. Animals 70 and 73 were coinfected with a *B. krasnovii*-like isolate (ML70XJBT) and an isolate of an unknown genotype (MM73XJBT) (Table 2).

**FIG 2:**
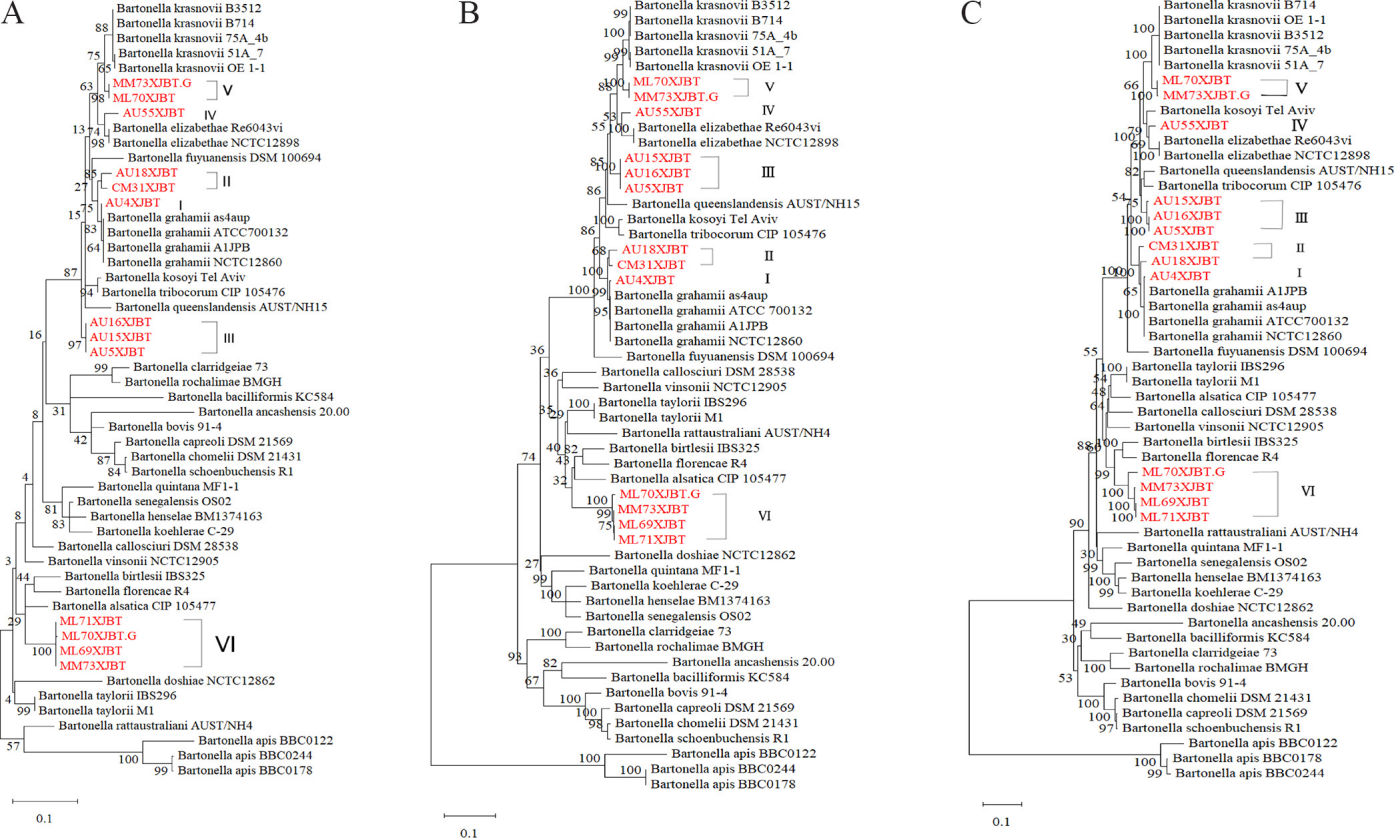
Phylogenetic trees based on 320-bp *gltA* genes (A), full-length *gltA* sequences (B), and 2,903-bp *rpoB* genes (C) of the *Bartonella* strains tested. The phylogenetic tree was constructed using Mega_X_10.1.8. Tree building was performed using the maximum likelihood (ML) method. Bootstraps were calculated with 1,000 replicates.

To further verify the relationship of the *Bartonella* isolates from Beitun rodents with recognized species of *Bartonella*, we performed phylogenetic analyses based on the full-length *gltA* and *rpoB* genes. In the phylogenetic trees of the sequences (Fig. 2), 13 *Bartonella* sequences were distributed into six clades. The *gltA* and *rpoB* sequences obtained in this study were all clustered into the clade of *Bartonella* species associated with rodents. AU4XJBT, AU18XJBT, and CM31XJBT were placed into the same phylogenetic group as B. grahamii as4aup. However, in the phylogenetic tree constructed by using the 2,903-bp *rpoB* gene, the bootstrap value between AU4XJBT, AU18XJBT, and CM31XJBT was 65%, which could not firmly indicate that the three strains belonged to the same species (Fig. 2C). Therefore, AU4XJBT and the latter two strains were artificially classified into cluster I and cluster II, respectively. Cluster III consisted of AU5XJBT, AU15XJBT, and AU16XJBT. Cluster IV was composed of AU55XJBT, which is closest to *B. elizabethae*. ML70XJBT and MM73XJBT.G were classified as belonging to cluster V. The four remaining strains (ML69XJBT, ML70XJBT.G, ML71XJBT, and MM73XJBT) were divided into cluster VI. By comparing the topological structures of the three evolutionary trees, the *Bartonella* isolates obtained in this study were divided into six clusters, but there were differences in the details of the evolutionary trees (Fig. 2A to C). To clarify the *Bartonella* species to which the 13 strains belonged, we studied the genomes of the *Bartonella* strains obtained in this study.

### Genomic features of the genus *Bartonella*.

The genomic features of these strains, including the G+C content, genome size, and number of coding sequences (CDSs), are presented in Table 3. Briefly, the G+C content of the genomes of these strains ranged from 37.68% to 38.93%, with an average of 38.24%. The average genome size of the 13 *Bartonella* strains in this study was 2.50 Mb (range, 2.14 to 2.89 Mb), with numbers of CDSs ranging from 1,998 to 2,347. All of the strains obtained in this study contained one predicted tmRNA (transfer-messenger RNA), but the predicted number of tRNAs was between 39 and 44, and the predicted number of rRNAs was between 3 and 4. The number of rRNAs predicted for CM31XJBT alone was 4 (Table 3). Under a threshold of 85%, the numbers of core genes and variable genes identified by pangenomic analysis of 52 strains (13 in this study and 39 in previous publications) were 129 and 18,446, respectively. Core-genome single-nucleotide polymorphism (SNP) analysis was carried out using the snp-sites command. A total of 20,102 SNP sites were selected from the core genome, and these sites were integrated for the construction of the SNP evolutionary tree.

**TABLE 3:** General features of sequenced *Bartonella* species in this study

Sample ID	No. of scaffolds	Genome size (Mb)	Sequence % GC content	*N*_50_ (Mb)	Total length of CDS (Mb)	No. of CDSs	No. of rRNAs	No. of tRNAs	No. of tmRNAs
AU5XJBT	79	2.44	38.4	12.06	1.7	2,266	3	39	1
AU15XJBT	114	2.81	38.34	10.92	1.74	2,347	3	39	1
AU16XJBT	82	2.5	38.39	16.15	1.69	2,220	3	39	1
AU4XJBT	39	2.48	37.82	11.12	1.58	1,998	3	39	1
AU18XJBT	161	2.89	37.68	5.82	1.68	2,223	3	39	1
CM31XJBT	85	2.32	37.88	9.26	1.72	2,250	4	43	1
AU55XJBT	38	2.14	38.39	13.67	1.75	2,135	3	43	1
ML69XJBT	148	2.56	38.54	6.46	1.67	2,237	3	39	1
ML70XJBT.G	85	2.46	38.93	10.39	1.78	2,294	3	44	1
ML71XJBT	149	2.53	38.58	6.77	1.65	2,209	3	39	1
MM73XJBT	124	2.65	38.47	8.27	1.59	2,095	3	39	1
ML70XJBT	110	2.28	37.87	7.52	1.63	2,108	3	41	1
MM73XJBT.G	70	2.41	37.85	13.94	1.65	2,109	3	40	1

### Genomic analysis.

**FIG 3:**
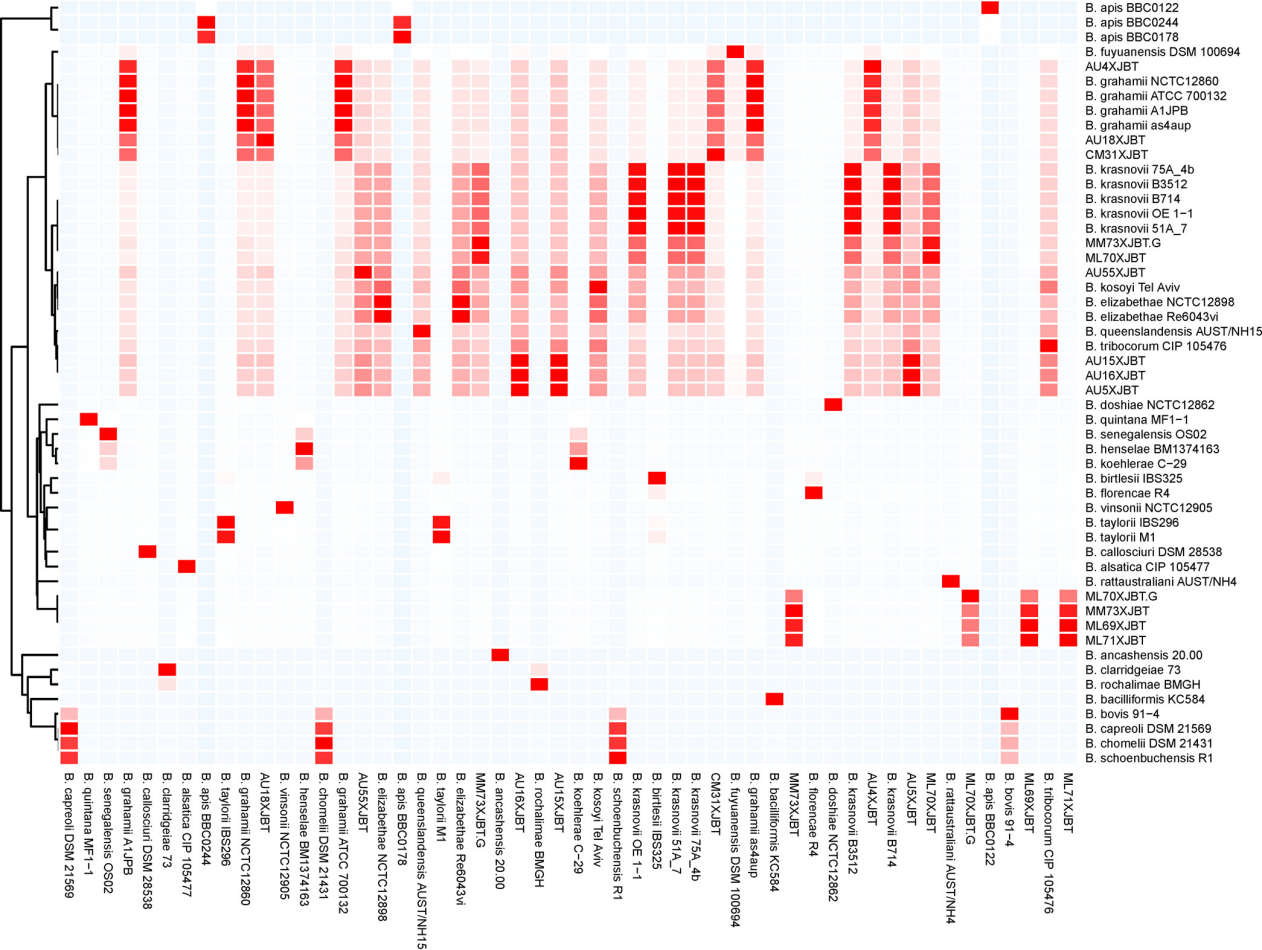
Heatmap of pairwise ANI values for 52 type strains. The values underlying this heatmap are provided in Table S1 in the supplemental material.

The average nucleotide identity (ANI) values of the 13 strains obtained in this study and 39 strains downloaded from the National Center for Biotechnology Information (NCBI) database for the genus *Bartonella* were used to assess the overall genome similarity (Fig. 3; see also Table S1 in the supplemental material). The current standard for a strain to be considered to belong to the same species is an ANI of ≥95% to 96% (11–13). The examined members of the *Bartonella* genus were found to have ANI values of >74.41%. Eight sets of synonymous species were identified based on the cutoff values for species delineation using the ANI (95%). AU4XJBT showed an ANI of >97%, compared to B. grahamii, which had the highest value (97.85%) found. The whole-genome ANI of AU5XJBT was calculated in relation to phylogenetically related species: ANI values of 99.55% and 99.99% were found compared to AU15XJBT and AU16XJBT, respectively. The three strains belonged to the same species and had the highest ANIs with *B. tribocorum* strain CIP 105476 (93.31%, 93.29%, and 93.30%, respectively). Similarly, ML70XJBT and MM73XJBT.G belonged to the same species (the ANI value was 98.49%), and ML69XJBT belonged to the same species as ML71XJBT and MM73XJBT (the ANI values were 99.81% and 98.39%, respectively). AU55XJBT and *B. elizabethae* had the highest ANI (93.22%) compared with the other *Bartonella* species analyzed. AU18XJBT and CM31XJBT had the highest ANIs (94.60% and 94.44%) with B. grahamii, respectively (Table S1).

**FIG 4:**
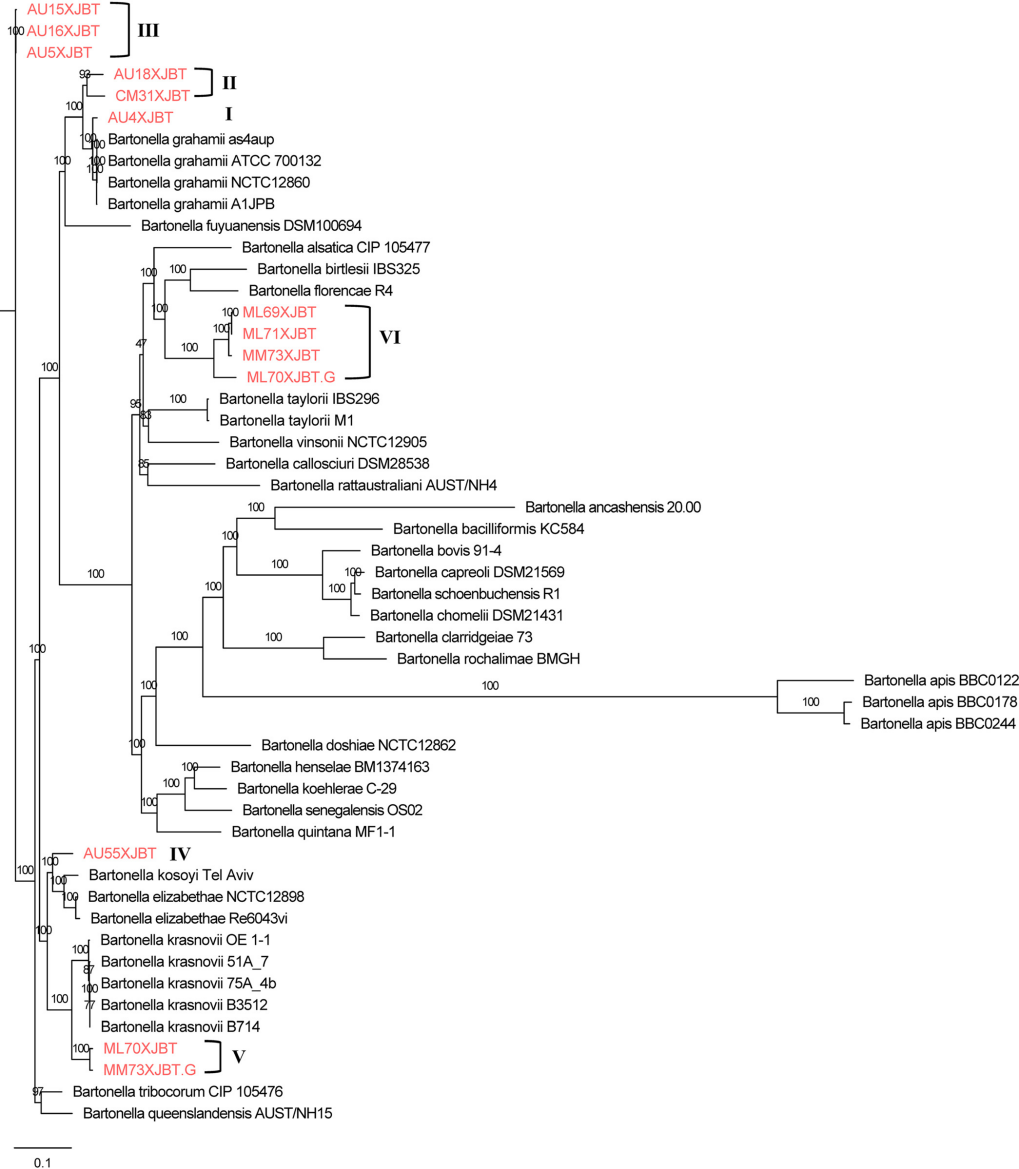
Core-genome SNP-based phylogenetic tree analysis of 52 *Bartonella* strains. Core is defined as a gene being present in at least 99% of samples, which allows some assembly errors in very large data sets. The phylogenetic tree was inferred by using the maximum likelihood method based on the general time-reversible model with 1,000 bootstrap replicates. Bootstrap values are indicated on the nodes.

We used phylogenomic analysis to examine the evolutionary relatedness among the 13 *Bartonella* strains in this study and 39 other *Bartonella* strains (Fig. 4). The genomic assembly quality control of the strains used to construct the phylogenetic tree met the requirements (genomes with *N*_75_ values of ≥10,000 bp and ≤500 undetermined bases per 100,000 bases) (14). Fewer SNPs were observed for core genes (129) than for total genes (18,575). The phylogenetic tree based on SNPs showed that AU4XJBT was clustered with B. grahamii. Three strains isolated from *A. uralensis* (AU5XJBT, AU15XJBT, and AU16XJBT) were clustered. AU55XJBT from *A. uralensis* had the highest similarity to *B. elizabethae* and *B. kosoyi*. The *B. krasnovii* clade included ML70XJBT from *M. tamariscinus* and MM73XJBT.G from *M. meridianus*. This analysis, based on SNPs in genomic sequences, showed that the ML69XJBT, ML70XJBT.G, ML71XJBT, and MM73XJBT isolates from *Meriones* were grouped (Fig. 4). We observed 13 *Bartonella* strains in this study, which were divided into six clusters using core-genome phylogenetic analysis; this was similar to the topology of the phylogenetic trees constructed with the *gltA* genes and *rpoB* genes.

## DISCUSSION

In Beitun and surrounding areas, six species of *Bartonella* were detected in four rodent species. Among them, B. grahamii was reported to be able to cause neuroretinitis in humans (15) and bilateral retinal artery branch occlusions (16). *B. tribocorum* has been reported in fleas of domestic dogs (17) and was implicated as the causative agent of human infections (18). In this study, the prevalence of *Bartonella* species in infected rodents varied from 0 to 50.0%, and the highest rate was detected in *M. meridianus*. Two or more *Bartonella* species were detected in *A. uralensis*. At least two species of *Bartonella* were isolated from both *M. tamariscinus* and *M. meridianus*. A B. grahamii-like isolate was detected in both the liver and spleen of a gray hamster. There was no statistically significant differences in the prevalences of *Bartonella* infection among the various rodents. According to the sequencing of the 320-bp *gltA* gene fragment, two strains from *A. uralensis* were more than 97% identical to B. grahamii, and one strain from *C. migratorius* was 99.69% similar to B. grahamii (Table 2). These results suggested that there might be intraspecies variations in B. grahamii from different host species; a similar phenomenon was previously reported on Heixiazi Island in northeastern China (19). In this study, we found that rodents 70 and 73 were coinfected by two *Bartonella* species using culture experiments. This suggests that we need to pay attention to this phenomenon in follow-up pathogen surveillance to prevent and control *Bartonella* disease better.

In this study, the species of *Bartonella* isolated were confirmed at both the gene and genome levels. Both gene-based phylogenetic analysis and SNP-based phylogenetic trees showed that the 13 *Bartonella* strains in this study were divided into 6 species (Fig. 2 and 4). However, in different phylogenetic trees, the closest known *Bartonella* species (in the NCBI database) of the 13 strains isolated in this study were different, especially those with ANI values of <95%. In general, an ANI value of <95% is used as a cutoff point for determining new species (11, 12, 20). In the ANI analysis of the genomic sequences of the *Bartonella* strains, all strains isolated from Xinjiang had ANI values of <95% compared to the *Bartonella* sequences in GenBank, except for the AU4XJBT strain. A previous study from Brazil reported that most *Bartonella* species showed ANI values of between 75% and 90%, and a cutoff point of 95% < ANI < 97% for the classification of new *Bartonella* subspecies was proposed (21). According to the existing ANI threshold, AU18XJBT and CM31XJBT do not belong to the same species. Similarly, ML70XJBT.G and the other three strains in cluster VI are also not the same species. Some inconsistencies were observed between these results and the phylogenetic trees based on the *gltA* gene, the *rpoB* gene, and the core-genome SNPs. On the one hand, the reasons might be the diversity and complexity of the *Bartonella* genome (18,446 variable genes). On the other hand, it may be due to gaps in the assembled genome. More *Bartonella* sequences are needed to verify whether there are different results of the phylogenetic analyses of the draft genome and the complete genome.

In this study, we could not accurately identify four strains of *Bartonella* (ML69XJBT, ML70XJBT.G, ML71XJBT, and MM73XJBT). They were highly similar to “*Candidatus* Bartonella gerbillinarum” (99.69% to 100%) by 320-bp *gltA* gene alignment. Comparison of the full-length *gltA* genes (1,296 bp) showed that B. alsatica had the highest similarity (90.28% to 90.51%) with these strains. B. taylorii had the highest similarity (91.04% to 91.26%) with these strains by comparison of the full-length *rpoB* genes (4,152 bp). All values were below the threshold of 97%. Phylogenetic trees based on SNPs in the core genome showed that the four strains mentioned above were closest to *B. florencae* R4. At the whole-genome level, the four strains had the highest ANIs (85.95% to 86.12%) with *B. florencae* R4, which did not reach the cutoff value that could accurately identify the species. These results indicate that ML69XJBT, ML70XJBT.G, ML71XJBT, and MM73XJBT may potentially be new species of *Bartonella*. The ANI values of ML70XBT.G and the other three strains were between 93.65% and 93.76%, suggesting that ML70XBT.G might be a subspecies.

In summary, based on the isolation results, the livers and spleens of infected animals had similar *Bartonella* burdens. These and previous observations suggest that the long-term persistence of *Bartonella* in these tissues may damage these organs (22). Comparative core-genome SNP, ANI, and gene analyses confirm the taxonomic placement of the *Bartonella* strains isolated in this study with other *Bartonella* spp. This study demonstrated the existence of multiple *Bartonella* infections in rodents in Beitun, Xinjiang. At the same time, *Bartonella* coinfection was found in rodents in this region. We suggest that more attention should be paid to coinfection in rodent-associated *Bartonella* surveillance in order to better understand the prevalence and genetic diversity of *Bartonella* species in rodents.

## MATERIALS AND METHODS

### Specimen collection and identification of rodents.

Rodents were captured using the trap-at-night method in June 2018 in Beitun and surrounding areas of the Xinjiang Uygur Autonomous Region (longitude, 87°53′ to 87°97′; latitude, 47°27′ to 47°57′). Traps baited with peanuts were set up in three regions consisting of five habitats, an *Elaeagnus angustifolia* plantation, a shelterbelt forest, tamarisk trees, grassland, and a semidesert. Sampling regions included Altay City, Beitun City, and Fuhai County, including four sampling sites (Fig. 1). Morphological and DNA barcoding techniques were used to identify the species of the trapped animals. Animals were accurately identified by the amplification of the mitochondrial cytochrome *c* oxidase subunit I (COI) gene with the universal primers shown in Table 4 (23). The collection time, site, habitat, species, gender, weight, head-body length, and tail length were recorded. The liver and spleen were collected into 1.5-mL Nunc CryoTubes and stored in liquid nitrogen prior to processing and laboratory testing.

**TABLE 4:** Oligonucleotide primers used for PCR

Target gene	Primer	Primer direction	Primer sequence (5′–3′)	Product length (bp)
*gltA*	BhCS.781p	Forward	GGGGACCAGCTCATGGTGG	379
	BhCS.1137n	Reverse	AATGCAAAAAGAACAGTAAACA	

COI	BatL5310	Forward	CCTACTCRGCCATTTTACCTATG	657
	R6036R	Reverse	ACTTCTGGGTGTCCAAAGAATCA	

### Isolation and purification of *Bartonella* organisms.

Approximately 10 mg of organ samples (liver or spleen) was added to 2-mL centrifuge tubes with 100 μL of tryptic soy broth (TSB) (BD) and some 1.2-mm grinding beads and disrupted using a Dounce-Potter homogenizer under sterile conditions, and the suspensions were inoculated onto tryptic soy agar (TSA) (BD) plates containing 5% defibrinated sheep blood. Plates were kept in a humid environment with 5% CO_2_ at 36.5°C for 20 days. Next, we selected suspected *Bartonella* organisms for purification and passage 2 to 4 times.

Material from a single colony was suspended in 100 μL of deionized water, heated for 30 min at 100°C, and cleared by centrifugation for 5 min at 6,000 × *g* at 4°C. The supernatant was used as a DNA template for PCR. The initial screening of *Bartonella* DNA was performed using PCR to detect a portion of the citrate synthase gene (*gltA*) with the primers BhCS.781p and BhCS.1137n (Table 4), as described previously (24). The pure bacterial cultures were collected in brain heart infusion (BHI) broth (BD) supplemented with 30% glycerol at −70°C (19, 25).

### Genome sequencing, assembly, and annotation.

To further characterize the positive samples in this study, genomic DNA from positive samples was extracted using the Wizard genomic DNA purification kit (Promega, Madison, WI, USA) according to the manufacturer’s instructions. Genomic DNA was extracted by using SDS (26), followed by agarose gel electrophoresis to determine the purity and integrity of the DNA, and quantification was performed by using a Qubit 2.0 fluorometer. DNA samples quantified by electrophoresis were randomly interrupted by using a Covaris ultrasonic breaker at a length of about 350 bp. After the processing of complete DNA fragments, the entire library preparation was completed using the NEBNext Ultra DNA library prep kit for Illumina (New England BioLabs, USA). The Qubit 2.0 instrument was used for the initial quantification of the library, the library was diluted to 2 ng/μL, and the inserts in the library were subsequently detected using an Agilent 2100 instrument. Finally, the effective concentration of the library was accurately quantified by using a quantitative PCR (qPCR) method to ensure the quality of the library. After library inspection, different libraries were sequenced on the Illumina NovaSeq PE150 (150-bp paired-end) platform according to the effective concentration and the target amount of data. All sequencing depths were about 100-fold. Readfq (version 10) was used to filter the raw data and obtain effective data (clean data) for downstream analysis. The specific processing steps were as follows: (i) reads with >40% low-quality bases (mass value of ≤20) were filtered out, (ii) reads containing >10% N bases were filtered out, (iii) reads with overlaps and adapter sequences exceeding a certain threshold (the default was 15 bp) were removed, and (iv) host-derived reads and duplications were filtered out. All good-quality paired-end reads were then *de novo* assembled using SPAdes v3.8.0 (27). The sequenced genomes were annotated using Prokka v1.14.5 (28).

### Phylogenetic analysis of the *gltA* and *rpoB* genes.

The DNAStar Lasergene SeqMan (plug-in in the DNAStar installation package) procedure was applied for sequence splicing, and a BLAST search was performed (http://blast.ncbi.nlm.nih.gov/Blast.cgi) to determine splicing sequence similarity values. *Bartonella* isolates were considered similar to validated *Bartonella* species if the *gltA* fragments had ≥97% sequence similarity (29). The full-length *gltA* genes and *rpoB* genes of the *Bartonella* strains cultured in this study were screened from whole-genome sequences using localized BLAST analysis (ncbi-blast-2.13.0+). Gene sequences were aligned using ClustalW. A phylogenetic tree was created by using the maximum likelihood (ML) method in Mega_X_10.1.8 with 1,000 bootstrap replicates. The best evolutionary model was selected by the Models program in Mega_X_10.1.8.

### Phylogenomic analyses.

Pairwise average nucleotide identity (ANI) values were estimated using FastANI v1.33 (12). The results were visualized and plotted with the R package pheatmap. The assembled *Bartonella* isolate genomes (this study) were compared with 39 published *Bartonella* genomes with valid names retrieved from the National Center for Biotechnology Information (NCBI) RefSeq database (30), but some *Bartonella* genomes were not included in the RefSeq database (Tables 2 and 5). At the same time, the core genomes and pangenomes of the *Bartonella* genomes mentioned above were compared. Single nucleotide polymorphisms (SNPs) in the core genomes were found using Roary v1.13.0 (31) analysis of these genomes. The minimum percent identity by BLASTp for Roary software was set to 85%. These SNPs were aligned with MAFFT (32) and were later used for phylogenetic analyses. The phylogenetic tree was inferred with IQ-TREE v2.1.2 by using the ML method. The best model was selected by the ModelFinder program. The number of bootstrap replicates was 1,000.

**TABLE 5:** *Bartonella* species from the NCBI selected for SNP and ANI analyses^*a*^

Species	GenBank accession no.	Genome size (Mb)	No. of scaffolds	Sequence % GC content	No. of CDSs	Genome coverage (×)	Source
Bartonella alsatica CIP 105477	NZ_CP058235	1.66	1	36.9	1,340	1,053	*Oryctolagus cuniculus*
Bartonella ancashensis 20.00	NZ_CP010401	1.47	1	38.4	1,242	42	Human (Homo sapiens)
Bartonella apis BBC0122	NZ_CP015625	2.91	1	45.7	2,393	150	Apis mellifera
Bartonella apis BBC0178	NZ_CP015820	2.60	1	45.3	2,190	50	Apis mellifera
Bartonella apis BBC0244	NZ_CP015821	2.64	1	45.2	2,241	75	Apis mellifera
Bartonella bacilliformis KC584	NZ_CP045671	1.41	1	38.2	1,177	500	Human (Homo sapiens)
Bartonella birtlesii IBS 325	NZ_CM001557	1.83	1	37.9	1,552	27.7	Mouse (*Apodemus* spp.)
Bartonella bovis 91-4	NZ_CM001844	1.62	1	37.4	1,373	120	Cat (Felis catus)
Bartonella callosciuri DSM 28538	NZ_JACHIM000000000	1.75	36	36.0	1,519	609	Plantain squirrel (*Callosciurus notatus*)
Bartonella capreoli DSM 21569	NZ_CADDZX000000000	1.81	439	37.8	1,818	NA	NA
Bartonella chomelii DSM 21431	NZ_JACJIR010000001	1.57	33	37.5	1,321	957	NA
Bartonella clarridgeiae 73	NC_014932	1.52	1	35.7	1,228	NA	Cat (Felis catus)
Bartonella doshiae NCTC12862	NZ_JH725094	1.81	7	38.0	1,543	160	Vole (*Microtus agrestis*)
Bartonella elizabethae NCTC12898	NZ_LR134527	2.02	1	38.4	1,659	NA	NA
Bartonella elizabethae Re6043vi	NZ_JH725139	1.96	8	38.4	1,587	284	Polynesian rat (*Rattus exulans*)
Bartonella florencae R4	NZ_HE997451	2.05	89	38.7	1,786	36	*Crocidura russula* shrew
Bartonella fuyuanensis DSM 100694	NZ_JACIFE010000001	1.94	81	36.7	1,594	771	Field mouse (Apodemus agrarius)
Bartonella henselae BM1374163	NZ_HG965802	1.91	1	38.2	1,566	NA	Human (Homo sapiens)
Bartonella koehlerae C-29	NZ_KL407334	1.75	3	37.6	1,408	169	Cat (Felis catus)
*Bartonella kosoyi* Tel Aviv	NZ_CP031843	2.23	2	38.5	1,898	100	Rodent (*Rattus rattus*)
Bartonella queenslandensis AUST/NH15	NZ_HE997969	2.38	598	38.9	2,127	NA	Wild rat (*Rattus leucopus*)
Bartonella quintana MF1-1	NZ_AP019773	1.59	1	38.8	1,285	878	Macaque (Macaca fuscata)
Bartonella rattaustraliani AUST/NH4	NZ_CALW02000108	2.16	28	38.8	1,850	NA	Wild rat (*Rattus tunneyi*)
Bartonella rochalimae BMGH	NZ_KL407337	1.53	3	35.9	1,284	165	Human (Homo sapiens)
Bartonella schoenbuchensis R1	NZ_CP019789	1.73	2	37.8	1,537	45	*Capreolus capreolus*
Bartonella senegalensis OS02	NZ_HE997540	2.00	41	38.8	1,614	30	Soft tick (*Ornithodoros sonrai*)
Bartonella taylorii IBS296	NZ_CP083444	1.95	1	38.8	1,660	200	Mouse (*Microtus* sp.)
Bartonella taylorii M1	NZ_CP083693	1.94	1	38.7	1,618	200	*Apodemus sylvaticus*
Bartonella tribocorum CIP 105476	NC_010161	2.64	2	38.9	2,260	NA	Wild rat (Rattus norvegicus)
Bartonella vinsonii NCTC12905	NZ_LR134529	2.00	1	39.1	1,750	NA	NA
*Bartonella krasnovii* OE 1-1	NZ_CP031844	2.19	2	38.2	1,789	100	Flea
*Bartonella krasnovii* B3512	NZ_CP093033	2.07	1	37.9	1,644	100	Rodent
*Bartonella krasnovii* B714	NZ_CP093034	2.05	1	37.9	1,628	100	Rodent
*Bartonella krasnovii* 51A_7	NZ_CP093043	2.18	1	38.2	1,792	100	Flea
*Bartonella krasnovii* 75A_4b	NZ_CP093042	2.1	1	37.9	1,694	100	Flea
Bartonella grahamii as4aup	NC_012846	2.34	2	38.1	1,956	NA	*Apodemus sylvaticus*
Bartonella grahamii ATCC 700132	NZ_JACX01000001	2.2	66	38	1,822	NA	NA
Bartonella grahamii A1JPB	NZ_CACVBJ010000001	2.49	472	38	2,331	54	NA
Bartonella grahamii NCTC12860	NZ_UFTD01000005	2.39	5	38.2	2,030	NA	Bank vole (*Myodes glareolus*)

a NA, not available.

### Coinfection certification experiment.

When the *gltA* gene was used for the preliminary identification of *Bartonella* species obtained in this study, an interesting phenomenon was found: different species of *Bartonella* were cultured from the spleen and liver tissues of animals 70 and 73. To verify the presence of two *Bartonella* coinfections in rodents 70 and 73, tissue samples from these two animals were recultured. In this *Bartonella* isolation experiment, we selected as many monoclonal bacteria as possible from the primary culture medium for passage culture. Next, the appropriate numbers of colonies were selected from the purified culture medium to prepare a DNA template for PCR, and the *gltA* gene was then amplified for identification.

### Statistical analysis.

R v4.1.2 was used for statistical analysis, with the significance level set at a *P* value of 0.05.

### Downloading of publicly available assemblies.

The assembly genome sequences of validly named *Bartonella* species were downloaded from the NCBI public database on 1 July 2022. All publicly available assemblies were subjected to quality control by using Quast v5.0.2 (33). Genomes with *N*_75_ values of <10,000 bp and >500 undetermined bases per 100,000 bases were discarded (14). Table 5 shows the *Bartonella* species downloaded from the NCBI that were included in this genomic analysis.

### Data availability.

The novel genome sequences of the *Bartonella* strains have been submitted to the NCBI GenBank database under accession numbers JAQNDQ000000000, JAQITG000000000, JAQITF000000000, JAQNDP000000000, JAQITE000000000, JAQITC000000000, JAQITD000000000, JAQITB000000000, JAQISX000000000, JAQITA000000000, JAQISZ000000000, JAQISY000000000, and JAQISW000000000.
